# Mortality in a cohort of WTC-exposed law-enforcement officers compared to non-WTC law-enforcement officers

**DOI:** 10.1007/s00420-025-02121-2

**Published:** 2025-02-01

**Authors:** Ankura Singh, Malak Khalifeh, John Violanti, Rachel Zeig-Owens, Andrew C. Todd, Moshe Z. Shapiro, Madeline E. Carwile, Christopher R. Dasaro, Jiehui Li, Janette Yung, Mark R. Farfel, Robert M. Brackbill, James E. Cone, Baozhen Qiao, Maria J. Schymura, David J. Prezant, Charles B. Hall, Paolo Boffetta

**Affiliations:** 1 Department of Medicine, Montefiore Medical Center, Bronx, NY USA; 2 Fire Department of the City of New York, Brooklyn, NY USA; 3 Stony Brook Cancer Center, Stony Brook University, Stony Brook, NY 11794 USA; 4 Department of Epidemiology and Environmental Health, School of Public Health and Health Professions, University at Buffalo, State University of New York, Buffalo, NY USA; 5 Department of Epidemiology and Population Health, Albert Einstein College of Medicine, Bronx, NY USA; 6 Department of Environmental Medicine and Climate Science, Icahn School of Medicine at Mount Sinai, New York, NY USA; 7 New York City Department of Health and Mental Hygiene, World Trade Center Health Registry, Long Island City, NY USA; 8 New York State Department of Health, Bureau of Cancer Epidemiology, Albany, NY USA; 9 Department of Family, Population and Preventive Medicine, Renaissance School of Medicine, Stony Brook University, Stony Brook, NY USA; 10 Department of Medical and Surgical Sciences, University of Bologna, Bologna, Italy

**Keywords:** Law-enforcement officers, Mortality, Occupational health, Epidemiology

## Abstract

**Purpose:**

World Trade Center (WTC) rescue/recovery workers were exposed to materials hazardous to health. Previous studies found lower than expected mortality among WTC rescue/recovery workers when compared to general populations, possibly due to healthy worker effects, better healthcare access and/or incomparability of the groups. We compared mortality rates in WTC-exposed law enforcement officers (LEOs) with rates in LEOs employed by the Buffalo, NY, Police Department. We also compared both cohorts to the general population.

**Methods:**

Follow-up began at the later of one year after enrollment date or 1/1/2005 and ended at the earlier of death date or 12/31/2018. Analyses were restricted to ages 40–79 years (N = 11,476 WTC LEOs, N = 1668 non-WTC LEOs). We estimated standardized mortality ratios (SMRs) in each cohort using stratum-specific US mortality rates. Relative rates (RRs) and 95% CIs were estimated for the WTC vs. the Buffalo cohort using Poisson regression models adjusted for sex, race, age-group, and calendar-period.

**Results:**

185 deaths were observed in the WTC cohort and 186 in the Buffalo cohort. All-cause and cause-specific SMRs were significantly lower in the WTC cohort. Similarly, the adjusted all-cause mortality RR for the WTC vs. Buffalo cohorts was 0.30 (95% CI = 0.23–0.40). The cause-specific mortality RRs were all significantly < 1.

**Conclusion:**

We found lower overall and cause-specific mortality rates in WTC LEOs compared with both the general population and Buffalo LEOs. These results suggest that factors other than healthy worker effects, such as access to healthcare via the WTC Health Program, contribute to lower mortality rates in WTC rescue/recovery workers.

## Introduction

The World Trade Center (WTC) attacks on September 11, 2001 (9/11) exposed law enforcement officers (LEOs) and other rescue/recovery workers to many toxic and carcinogenic particles, including heavy metals, asbestos, benzene, polycyclic aromatic hydrocarbons and polychlorinated biphenyls (Lioy et al. 2002; Landrigan et al. 2004; Lioy and Georgopoulos 2006). Studies have reported that WTC exposure was associated with some cancers and cardiovascular, respiratory, and gastric diseases, as well as psychosocial factors such as depression, post-traumatic stress disorder, smoking and alcohol abuse (Herbert et al. 2006; Ekenga and Friedman-Jimenez 2011; Perlman et al. 2011; Soo et al. 2011; Wisnivesky et al. 2011; Goldfarb et al. 2021a, b, c; Webber et al. 2021), indicating that WTC-exposed workers may be at higher risk for mortality.

However, previous studies have reported that all-cause mortality and mortality from several major causes were significantly lower than expected in individual WTC cohorts compared to US or New York State (NYS) populations, as well as in a pooled cohort of WTC responders (Jordan et al. 2018; Colbeth et al. 2020; Li et al. 2022). One explanation for the lower mortality rates may be bias from the healthy worker effect, in which occupational groups, by virtue of their inclusion in employment, are healthier than the general population (Li and Sung 1999). Another explanation, also related to the healthy worker effect, is that compared to general populations, the WTC rescue/recovery workers have better access to healthcare due to their employment benefits. Additionally, they are offered no-cost comprehensive medical monitoring and treatment via the WTC Health Program of major diseases/conditions deemed to be WTC exposure-related (Centers for Disease Control and Prevention 2023).

A possible approach to assessing the contribution of these factors in generating an apparent protective effect on the mortality of WTC rescue/recovery workers is to use a comparable reference population, which, except for the WTC experience, has had similar occupational exposures and pre-employment screening. In this approach, subgroups of WTC rescue/recovery workers belonging to specific occupational groups are selected and compared to similar workers with no known WTC exposure. One such study compared the mortality rates in WTC-exposed and non-WTC-exposed firefighters (Singh et al. 2023). In that study, mortality was lower in the WTC-exposed firefighters, suggesting factors beyond healthy worker effects, such as access to no-cost health monitoring and treatment for WTC Health Program-covered conditions.

LEOs comprised a large proportion of WTC rescue/recovery workers, including 21% of members of the WTC Health Registry (WTCHR) and 42% of members of the WTC General Responder Cohort (GRC) (Boffetta et al. 2016). For this study, we compared mortality rates in WTC-exposed LEOs with those in a cohort of non-WTC-exposed LEOs from Buffalo, NY (Vena et al. 1986; Violanti et al. 1998, 2021), as well as comparing mortality rates in each LEO cohort with those of the US general population.

## Methods

### Study population

The WTC LEO cohort was comprised of LEOs in the Combined WTC Rescue/Recovery Worker Cohort (Combined Cohort) who were enrolled in either the GRC or the WTCHR. These cohorts consist of both paid and volunteer rescue/recovery workers, including older/retired LEOs, who were involved in the WTC rescue/recovery effort anytime on or after 9/11/2001 (up until July 2002) (Brackbill et al. 2021). Details of the pooling and deduplication process that created the source population, the Combined Cohort, have been reported elsewhere (Brackbill et al. 2021). Previous mortality analyses in the Combined Cohort were restricted to WTC rescue/recovery workers who were ≥ 18 years old on 9/11/2001, had provided race/ethnicity information, and had enrolled in their respective WTC rescue/recovery worker cohorts prior to 1/1/2011 (Li et al. 2022). In the present study, we further restricted our analyses to LEOs who were White, Black or Hispanic and within the 40–79 years age range during the study period (1/1/2005–12/31/2018), due to small numbers in other age and race groups. Persons who died within the first year of enrollment or before 2005 were also excluded, leaving 11,476 WTC LEOs in the population for analyses.

The Buffalo LEOs included in this study were a subpopulation of a larger cohort assembled for a previous study of LEO mortality. This source population, constructed using Buffalo NY Police Department employment records, included officers who had worked at least five years for the Buffalo Police Department between 1/1/1950 and 12/31/2018 and were not missing birth date nor dates of employment (Violanti et al. 2021). For the current analyses, in order to be comparable with the WTC-exposed population, the non-WTC-exposed population was restricted to White, Black and Hispanic Buffalo cohort members who were employed on or after 1/1/2000, alive as of 1/1/2005, and fell within the 40–79 years age range during the study period (1/1/2005–12/31/2018) (N = 1668). Only grouped data were available for this cohort.

This study was approved by the Institutional Review Boards (IRB) of the Albert Einstein College of Medicine, New York City Department of Health and Mental Hygiene, New York State Department of Health, and State University of New York at Buffalo. IRBs of the Icahn School of Medicine at Mount Sinai and Stony Brook University granted exemptions.

### Death data

Linkage with the National Death Index (NDI) was performed independently for the GRC and WTCHR cohorts, and then pooled for analyses in the Combined Cohort (Li et al. 2022). We identified all deaths that occurred by 12/31/2018. Underlying causes of death were coded based on the International Classification of Disease codes, 10th revision (ICD-10) (Robinson et al. 2006) and provided by NDI. For the purpose of these analyses, ICD-10 codes were categorized into six major categories including all cancer mortality, respiratory cancer-related mortality, and cardiovascular, respiratory, digestive and external causes of mortality; these were derived from cause of death categories defined by the National Institute for Occupational Safety and Health (NIOSH) (National Institute for Occupational Safety and Health, Robinson et al. 2006). External cause mortality included deaths due to suicide, homicide, accidents and falls.

For the Buffalo cohort, sources of mortality data were the NDI (~ 95%), New York State Vital Statistics Division, benefit and pension programs of the city of Buffalo, the New York State Retirement System, Buffalo Police employment records, Buffalo Police Association publications, and obituaries. Death certificates were coded by state mortality coders according to the ICD revision in effect at the time of death (Violanti et al. 2021). Again, for the present analyses, ICD codes were re-coded into the same major cause of death categories listed above.

Lastly, we obtained overall and cause-specific US mortality rates, stratified by calendar period, sex, race, and age group, from NIOSH’s Life Table Analysis System 1960–2019 rate file (National Institute for Occupational Safety and Health, Bertke and Kelly-Reif 2022).

### Statistical analyses

Cohort demographic characteristics, including age, sex and race, were compared using descriptive statistics (proportions or means ± standard deviations [SD]). In the WTC and Buffalo cohorts, follow-up time began at the later of one year after participant enrollment date or 1/1/2005, and ended on death date or 12/31/2018, whichever occurred first. We used stratum-specific (calendar period-, sex-, race-, and age group-specific) US mortality rates to estimate standardized mortality ratios (SMRs) in each cohort, first multiplying the rates by the numbers of person-years in the corresponding strata for each cohort and then summing across strata to calculate expected death counts. The numbers of actual deaths observed in the WTC and Buffalo cohorts were each divided by the corresponding expected death counts to get the SMRs. Ninety-five percent confidence intervals (CIs) for the SMRs were estimated using Poisson assumptions (Byar’s approximation) (Breslow and Day 1987). Mortality relative rates (RRs) were then estimated for the WTC cohort with the Buffalo cohort as reference, using Poisson regression models for grouped data, adjusting for sex, age group (in 10-year strata), race (non-Hispanic White, non-Hispanic Black, and Hispanic), and calendar period (in five-year strata).

Analyses were conducted using SAS version 9.4 (SAS Institute Inc., Cary, NC, https://www.sas.com). All statistical analyses were two-sided and p values ≤ 0.05 were considered significant.

## Results

Both the WTC and Buffalo cohorts were mostly male (84.3% and 85.7%, respectively). The WTC cohort had a higher proportion of Hispanic members and lower proportions of non-Hispanic White and Black members than the Buffalo cohort (Table 1). The average age of WTC LEOs on 9/11/2001 was 36.8 years (SD ± 6.4), compared with 48.9 ± 13.6 years for Buffalo LEOs. There were 185 deaths among 11,476 WTC cohort members with 118,538 years of follow-up during the study period, and 186 deaths among 1668 Buffalo cohort members with 18,018 years of follow-up. In both cohorts, cancer and cardiovascular disease were the most common causes of death (Table 2).

**Table 1:** Characteristics of WTC-exposed and non-WTC-exposed law enforcement officers

	WTC-Exposed			Non-WTC-Exposed		
	Males	Females	Total	Males	Females	Total
Total	9675	1801	11,476	1430	238	1668
Age on 9/11 (years), Mean (SD)	36.8 (6.4)	36.7 (6.5)	36.8 (6.4)	50.5 (13.8)	39.1 (6.5)	48.9 (13.6)
Race						
Non-Hispanic white	6896 (71.3%)	762 (42.3%)	7658 (66.7%)	1156 (80.8)	160 (67.2)	1316 (78.9)
Non-Hispanic black	817 (8.4%)	488 (27.1%)	1305 (11.4%)	195 (13.6)	72 (30.3)	267 (16.0)
Hispanic	1962 (20.3%)	551 (30.6%)	2513 (21.9%)	79 (5.5)	6 (2.5)	85 (5.1)
Number of deaths	158	27	185	178	8	186
Person-years	100,311	18,227	118,538	15,339	2679	18,018

*WTC* World Trade Center, *SD* Standard Deviation

**Table 2:** Standardized Mortality Ratios (SMRs) of all-cause and cause-specific mortality in WTC-exposed and non-WTC-exposed law enforcement officers vs. US adults, 1/1/2005–12/31/2018

Cause of death (NIOSH major category)^a^	WTC-Exposed			Non-WTC-Exposed		
	N	SMR	95% CI	N	SMR	95% CI
All	185	0.28	0.24–0.33	186	0.87	0.75–1.00
All cancers (02, 03, 04, 05, 06, 07, 08, 09, 10)	65	0.41	0.31–0.52	66	1.02	0.79–1.30
Respiratory cancer (04)	12	0.29	0.15–0.50	18	0.87	0.52–1.38
Heart diseases and other diseases of the circulatory system (16, 17)	42	0.23	0.16–0.31	57	0.88	0.67–1.14
Diseases of the respiratory system (18)	≤ 5	0.15	0.05–0.34	18	0.92	0.55–1.46
Diseases of the digestive system (19)	11	0.26	0.13–0.46	7	0.69	0.28–1.43
External causes (24, 25, 26, 27)	38	0.34	0.24–0.47	12	0.71	0.37–1.24

*WTC *World Trade Center, *NIOSH* National Institute for Occupational Safety and Health, *CI* Confidence Interval

^a^International Classification of Diseases, 10th revision codes grouped into major cause of death categories (Robinson et al. 2006)

WTC LEOs had significantly lower-than-expected mortality when compared to demographically similar US adults (SMR = 0.28, 95% CI = 0.24–0.33) (Table 2). For all-cause mortality and for each type of cause-specific mortality that we assessed, SMRs were significantly below 1. Buffalo LEOs had modestly lower all-cause mortality than US adults, although the difference only bordered on statistical significance (SMR = 0.87, 95% CI = 0.75–1.00). Cancer-specific mortality, respiratory cancer-specific mortality, and cardiovascular disease-, respiratory disease-, digestive disease- and external cause-specific mortality were not significantly lower in the Buffalo cohort vs. the US population.

Analyses comparing mortality rates in the two cohorts showed that the rate of all-cause mortality was significantly lower among WTC vs. Buffalo LEOs, after controlling for calendar period, sex, race and age group (RR = 0.30, 95% CI = 0.23–0.40) (Table 3). Similarly, rates of mortality from all cancers, respiratory cancer, cardiovascular diseases, respiratory diseases, digestive diseases and external causes were all significantly lower in the WTC cohort.

**Table 3:** Adjusted relative rates (RRs) of all-cause and cause-specific mortality in WTC-exposed vs. non-WTC-exposed law enforcement officers, 1/1/2005–12/31/2018

Cause of death (NIOSH major category)^a^	RR^b^ (95% CI)
All	0.30 (0.23–0.40)
All cancers (02, 03, 04, 05, 06, 07, 08, 09, 10)	0.34 (0.21–0.56)
Respiratory cancer (04)	0.22 (0.07–0.65)
Heart diseases and other diseases of the circulatory system (16, 17)	0.35 (0.20–0.60)
Diseases of the respiratory system (18)	0.14 (0.03–0.60)
Diseases of the digestive system (19)	0.19 (0.06–0.60)
External causes (24, 25, 26, 27)	0.43 (0.19–0.99)

*WTC *World Trade Center, *NIOSH* National Institute for Occupational Safety and Health, *CI* Confidence Interval

^a^International Classification of Diseases, 10th revision codes grouped into major cause of death categories (Robinson et al. 2006)

^b^Adjusted for sex, race, 10-year age group, 5-year calendar period

## Discussion

This study found that WTC-exposed LEOs had lower mortality rates compared with both the US general population and an occupational comparison cohort of non-WTC-exposed LEOs from Buffalo, NY. Previous studies showed lower mortality rates among WTC rescue/recovery workers compared with the New York City, NY state and/or US general populations (Jordan et al. 2018; Colbeth et al. 2020; Li et al. 2022). Most recently, in a study of the Combined WTC Rescue/Recovery Worker Cohort, the overall mortality of all WTC rescue/recovery workers between 2002 and 2016 was approximately half of that of the corresponding NYS population and 57% lower than that of the corresponding US population (Li et al. 2022). The results of the present SMR analyses, limited to LEOs, show an even greater difference in mortality in the WTC-exposed cohort vs. the US population, with overall mortality over 70% lower than expected given US rates.

Some of the previously observed mortality benefit among WTC rescue/recovery workers may be attributable to the healthy worker effect, in which occupational cohorts experience lower mortality than general populations (Li and Sung 1999; Pearce et al. 2007). LEOs often need to meet certain health requirements to be hired, which may contribute to the healthy worker hire effect (Li and Sung 1999; Pearce et al. 2007; Chowdhury et al. 2017). The Buffalo LEO cohort also had lower than expected all-cause mortality when compared to the US general population; however, the difference was modest (13%) and only bordered on statistical significance. Since WTC LEOs had significantly lower mortality rates than even Buffalo LEOs, the healthy worker effect would not fully explain the reduction in mortality. Our results suggest the role of factors specific to the WTC cohort. In a similar occupational comparison analysis of 10,786 WTC-exposed Fire Department of the City of New York (FDNY) firefighters and 8813 non-WTC-exposed firefighters from Chicago, Philadelphia and San Francisco, WTC-exposed firefighters experienced lower mortality from all-causes (RR = 0.54; 95% CI = 0.49–0.59) and from most specific causes during the first 15 years post-9/11, compared to non-WTC firefighters (Singh et al. 2023).

Factors other than the healthy worker effect that may affect mortality rates in WTC rescue/recovery worker cohorts include members’ access to no-cost, comprehensive health monitoring and treatment for WTC Health Program covered conditions, even after retirement (Santiago-Colon et al. 2020; Centers for Disease Control and Prevention 2023). Previous studies evaluated the effect of WTC Health Program services on health problems, including cancer, aerodigestive disorders and mental health conditions (Azofeifa et al. 2021; Goldfarb et al. 2021a, b, c; Smith et al. 2021). Azofeifa et al. reported high rates of utilization of WTC Health Program monitoring and treatment services among enrolled rescue/recovery workers and survivors (Azofeifa et al. 2021). A study of cancer incidence in WTC-exposed and non-WTC-exposed firefighters noted that cancers tended to be diagnosed at younger ages and earlier stages in the WTC cohort than in the non-WTC cohort and the US population, possibly as a result of WTC Health Program-related screening (Webber et al. 2021). In another study, lower all-cause and cancer-specific mortality rates were observed among WTC Health Program enrollees with cancer than in a reference population of cancer patients from southern NYS (Goldfarb et al. 2021a, b, c).

This is the first study to compare mortality rates in WTC vs. non-WTC LEOs. However, this study has several limitations, including the lack of data on some potential confounding variables, such as comorbid conditions and body mass index (BMI). We also lacked data on certain behavioral factors related to mortality, such as smoking and alcohol use levels in the Buffalo cohort. An additional limitation was the age difference between the WTC and Buffalo cohorts; even after restricting our analyses to those aged between 40 and 79 years anytime during the study period, the average age on 9/11 of the WTC cohort was more than 10 years less than that of the Buffalo cohort. This age discrepancy would partially explain the lower death rates in the WTC vs. non-WTC LEOs, although we attempted to counter it by controlling for age when estimating RRs. The inclusion criteria were also slightly different for the two LEO cohorts: the Buffalo LEOs came from a source population that only included LEOs who had been employed for at least five years. For the WTC LEO cohort inclusion criteria, there was not a five-year minimum employment period because we did not have members’ employment dates, only their dates of enrollment in the WTCHP or WTCHR. LEOs who leave the workforce before five years of law enforcement employment could be less healthy than those who work ≥ 5 years (Arrighi and Hertz-Picciotto 1994; Siebert et al. 2001). This would potentially cause a healthy worker survivor effect/bias in the Buffalo LEO cohort, resulting in mortality rates that are lower than they would be otherwise. Even with this potentially healthier Buffalo cohort, we note that we observed significantly *lower* mortality rates in the WTC LEO cohort. A final minor limitation was that the death data for the Buffalo cohort were obtained from both NDI and other sources, while death data for the WTC cohort were obtained solely from NDI. More complete ascertainment of deaths in the Buffalo cohort could have biased the WTC vs. Buffalo RRs away from the null, i.e., showing a more protective effect of WTC cohort membership.

Despite these limitations, the study results were consistent with those of previous studies showing lower than expected mortality in WTC-exposed rescue/recovery worker cohorts. WTC-exposed LEOs had lower all-cause and cause-specific mortality rates even when compared with a similar occupational cohort, implicating factors beyond the healthy worker effect. Longer-term follow-up of WTC rescue/recovery worker cohorts is needed to validate our findings and assess mortality in more senior age groups and within the different sex and race groups.

### What is already known about this topic

- WTC-exposed rescue/recovery workers’ mortality was significantly lower than that of the US or New York State (NYS) populations, suggesting healthy worker effects and possibly effects of other protective factors, such as greater access to no-cost health monitoring and treatment for covered conditions.

### What this study adds

- We found significantly lower rates of all-cause and cause-specific mortality in WTC-exposed vs. non-WTC-exposed law enforcement officers. These results suggest that factors other than the healthy worker effect contribute to lower mortality rates among LEOs in the WTC cohort.

### How this study may affect research, practice or policy

- Comprehensive health monitoring and treatment programs such as the WTC Health Program likely play an important role in reducing mortality.

## Data Availability

Data are available upon reasonable request from the corresponding author once the request is approved by the Principal Investigators of the original cohorts and the steering committee for the combined cohort in accordance with the official data sharing plan.
